# Forceps-Rotation in Occipito-Posterior Positions of the Vertex*Read before the Obstetrical Society of Cincinnati.

**Published:** 1897-10

**Authors:** William Gillespie

**Affiliations:** Cincinnati, Ohio


### FORCEPS-ROTATION IN OCCIPITO-POSTERIOR POSITIONS OF THE VERTEX.*

By WILLIAM GILLESPIE, M.D.,

CINCINNATI, OHIO.

I recently had the pleasure of coming to your society as a visitor and hearing a paper read by Dr. Stewart, on forceps delivery in occipito-posterior positions of the vertex. While Dr. Stewart did not advocate forceps rotation, yet several members of the society took occasion to question its advisability. They did not go into the subject, however, sufficiently to state their objections plainly. As I was interested in the subject, had used the forceps several times to accomplish rotation and had been unable to find sufficiently good reasons for discontinuing the practice, I concluded to bring the subject up again, hoping to profit by the discussion. It is stated, on authority that I do not question, that all but four per cent. of occiput posterior positions of the vertex will rotate anteriorly if proper flexion is secured and time given for nature to mold the head for her purpose.

*Read before the Obstetrical Society of Cincinnati.

In looking up statistics, however, I was struck with the fact that all of them were taken from the older writers, and had been collected at a time when assistance to the parturient woman was never thought of till nature had completely exhausted her resources. I seriously doubt if any prominent obstetrician of the present day can give, from his own experience, the per cent. which nature can not rotate. Few men would subject a patient to hours or perhaps days of hard labor, and wait till nature had been completely exhausted before extending a helping hand. These statistics then are of no value, except to remind us that nature does attempt to rotate and we should always give her credit for very efficient help. Any careful observer has occasionally seen the occiput rotate anteriorly above the brim, in the cavity of the pelvis, and many times, just before the head sweeps over the perineum. It can usually be said that so long as the relative size of the child's head and the pelvic canal are not out of proportion, descent occurs naturally, but when resistance is met, nature endeavors to overcome the difficulty by rotating, and thus presenting a narrower diameter. This is a very valuable hint from nature as to the proper management of these cases. We shall follow the rule laid down by Simpson in dealing with this subject. "In occiput posterior positions the mechanism of the extraction of the head with forceps should be an exact imitation of the mechanism of the expulsion of the head by nature." No higher law can be laid down, and it needs only to be added, provided we can do this with the least possible danger to the mother and child.

When progress ceases, flexion being perfect, and nature does not seem able to rectify the position without exhaustion, it is our duty to render assistance. There are many ways of doing this, but we are only considering instrumental interference, which is always called in after failure of less active measures. Special care is called for in the application of forceps, and special care in using them after the application is made.

The head should always be grasped by its biparietal diameter when possible. This is easily accomplished with the head low in the pelvis, but when high in the cavity, it is sometimes impossible.

If compelled to grasp the head irregularly, the operator should remember two great dangers. First, his hold is necessarily insecure and traction must be made with great care to avoid slipping of the instruments. Second, if the handles are forcibly compressed, in order to retain the grasp necessary for forcible traction, there is danger of posterior rotation of the occiput. I once had this experience. The instrument slipping and the case demanding prompt delivery, I compressed the handles forcibly, and one blade grasping the forehead in front, and the other the occiput behind the center of the head, the forceps was converted into two levers and the occiput was rotated into the hollow of the sacrum. I was compelled to perforate before accomplishing delivery.

If the head has become wedged in the cavity of the pelvis, and cannot be dislodged with the hand or vectis, I think it would be wiser to rotate with the forceps, than run the risks of slipping and posterior rotation from violent traction and compression. Acting on this belief, I succeeded three years ago in delivering a living child after failing to effect elevation with the hand or descent by traction. The patient was a primipara aged twenty, giving birth to a large male child, O. R. P. After making traction as forcibly as I thought safe with an irregular application, I began slowly and without much force to attempt rotation. The head occupying the right oblique diameter with the occiput behind, the left blade was applied to the right side of the forehead, while the right blade secured a hold on the left side of the occiput. Proceeding slowly and with great care, on account of the edges of the blades endangering the soft parts, the head was soon brought into and a little past the transverse diameter. Removing the blades and reapplying them to the opposite oblique diameter, the head was rotated into the second position and delivery was completed. A careful examination with Sims' speculum failed to show any lesion in the vagina. I would not recommend this procedure except in cases of extreme difficulty, and only then, to one who possessed some little skill and much patience, for it is only by the exercise of great care, and proceeding slowly and without violence, that freedom from danger can be secured.

In most cases, however, the forceps can be applied to the bi-parietal diameter. We then proceed as follows: Pull downward and backward, exercising much greater care than in anterior positions, and continuing the backward traction longer. Even when the forceps grasp the head regularly in these cases, an examination of the child's head reveals the fact, that the tips of the blades are applied behind the ears, unless a forceps with excessive pelvic curve is used. As the head from this point back rapidly narrows, and as the distance between the posterior edges of the blades, in most forceps, is greater than that between the anterior, it follows that while the application seems regular, most of the force is exerted by the anterior edge of the blades, and any premature forward move of the handles will cause the posterior edges to plow up the recto-vaginal septum, if the forceps do not lose their hold entirely. Even when great care is used the forceps sometimes slowly slip backward. Therefore pull slowly and with greater care than in anterior positions, and examine frequently to see that the blades are still in proper position. The downward traction should be continued until the head rests upon the perineum. I usually continue this traction till the perineum is somewhat distended, and if the application has not been regular, adjust the forceps to the bi-parietal diameter. The blades are then loosened, and by depressing the handles are made to assume a position more nearly parallel with the long axis of the head. Closing the blades and lifting the handles, you now strongly flex the head, and bringing it down till the perineum bulges violently and the vulva is considerably dilated, you have the head in nature's favorite position for rotating. Grasp the forceps with one hand near the head, and making that a fixed point slowly and without force, move the handles in the arc of a circle, to the mother's left, in O. R. and P., to her right, in O. L. and P. No force is required for this rotation; after the movement is once started it requires to be resisted rather than assisted. You may now leave the case to nature or reapply the forceps, but I have usually delivered the head with the forceps in the reversed position. This is accomplished by grasping the forceps with the right hand, near the vulva, and with the handles resting under the forearm lift the head over the perineum; assisting or restraining its advance, while with the left hand I shell out the head. During the rotation and extraction, the blades are in exact contact with the head and no danger is run of cutting the mother with projecting edges. It is a close imitation of nature's method of delivering these cases and thus complies with the law quoted from Simpson. It remains to be seen whether we have accomplished it with the least possible danger to mother and child. The principal objection given by writers on obstetrics, so far as the child is concerned, is danger of fatally twisting the spinal cord. When we come to consider the large number of cases delivered this way by nature's unaided efforts, and by abdominal palpation demonstrate that the body of the child does not follow the head in its rotation, and yet no harm results to the child, the thought presents itself why should artificial rotation, if carefully executed, be more dangerous than natural rotation. We have positive proof, however, that this fear is more theoretical than practical in the experiments of Tarnier. "From the experiments on many cadavers of new-born infants, I have proved that when the head is twisted one-half the circumference, the shoulders being fixed, the motion does not alone occur at the atlo-axoid joint, but throughout the whole of the cervical, and a portion of the dorsal vertebra twisting spirally. In order to make the head thus rotate great force must be used, and yet careful dissection has failed to reveal the slightest lesions in the membranes or the spinal marrow. But, it may be said, if the vertebræ are twisted, the spinal cord must be compressed. To guard against this objection, I substituted for the cord a fluid column, connected with an external glass tube. Every compression of the canal caused the fluid to rise in the tube, and yet torsion of the head did not," etc.

I think these experiments prove conclusively that danger from injury to the spinal cord as a result of forceps rotation is purely imaginary. I would ascribe a much more serious effect to the prolonged and severe flexion necessary for nature to successfully deliver these cases. I have seen several cases of convulsions in the new-born, which I felt sure were due to cerebral apoplexy as a result of prolonged and violent flexion.

As we have considered that proper flexion has been secured before resort to forceps, it will not be necessary to consider forceps in the reversed position as recommended by Richardson of Boston.

The alternative usually recommended in place of forceps rotation is delivering the head face to pubes.

Grandin and Jarman, in their work on Obstetric Surgery, advise that as soon as the forehead comes under the pubes the handles of the forceps should be depressed and the head forcibly extended until the forehead escapes. Then with the forceps, or with two fingers in the rectum, the head is raised over the perineum. This is a violent procedure, and they add, "The perineum is almost certainly torn and should be repaired at once." They say later it is hard to resist the temptation to rotate, but we have only to remember the risks the mother would run from such a procedure and resist the temptation. As their procedure is so sure to cause serious laceration I can hardly conceive of more serious results occurring from rotation with the head in this position.

Pajot, many years ago, proposed a procedure differing from the above in that instead of producing extension as the first step, violent flexion was secured and the occiput raised over the perineum. The neck then becoming the fixed point with the perineum as fulcrum extension completed delivery. This violent flexion by bringing the soft top of the head against the pubes might endanger the cranial contents, and Charpentier, in commenting on it, says it almost certainly produces laceration of the perineum, and he therefore prefers to rotate with forceps when possible. That some cases can be delivered in this position without damage, I do not doubt. I have myself seen four unaided deliveries in which no tear took place. Three in multipara who had been previously torn and one primipara with large vulva and very lax pelvic floor. As the cases when I have used forceps, as above described, have shown no larger per cent. of lacerations than anterior positions, I have continued to follow the practice rather than follow established rule and do plastic surgery. As the danger to the child from torsion of the spinal column seems to be proven by Tarnier's experiments to be in the imaginations of those who fear it, I have laid it aside until at least I find as good reasons for accepting the theory as I now have for rejecting it.

